# Prospective evaluation of a decision support system providing advice on pressure support from states of over- and under-support

**DOI:** 10.1186/2197-425X-3-S1-A680

**Published:** 2015-10-01

**Authors:** S Spadaro, DS Karbing, CA Volta, SE Rees

**Affiliations:** University of Ferrara / Intensive Care Unit, Ferrara, Italy; Department of Health Science and Technology, University of Aalborg / Respiratory and Critical Care Group, Aalborg, Denmark; Morphology Surgery and Experimental Medicine, University of Ferrara / Intensive Care Unit, Ferrara, Italy

## Introduction

Providing appropriate pressure support (PS) is a balance of avoiding over-support with risk of muscle atrophy and prolonged weaning, and under-support with risk of patient discomfort and stress. The Beacon Caresystem (Mermaid Care, Denmark) advises on PS using physiological models of lung mechanics, respiratory drive, acid-base status and muscle function and clinical preference functions quantifying risk of muscle atrophy, patient stress, and lung trauma. Mathematical models are tuned to measurements allowing advice to be patient specific.

## Objectives

This study investigates the initial changes in pressure support from levels of over- and under-support.

## Methods

Six ARDS patients residing in an ICU in Ferrara, Italy, have currently been included for this preliminary analysis. Informed consent and ethical approval was obtained. System's advice was followed for an hour from states of over- and under-support defined as 150% and 50% of baseline PS or PEEP. Average and spread are reported as mean ± SD.

## Results

**Figure 1:**
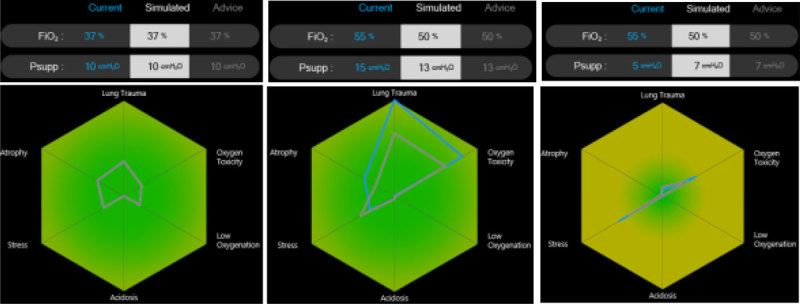
**Advice of Beacon Caresystem.**

SOFA score and age were 6.2 ± 1.9 and 71 ± 5 yrs, respectively. All patients were male. Four patients were subjected to PS changes and two to PEEP changes. Figure 1 illustrates response to over- and under-support as advice and preference functions in a patient where the system advised to return PS towards baseline. Average baseline PS was 10 ± 3 cm H_2_O. On average, Advice from PS150% and PS50% changed PS to 13 ± 4 cm H_2_O (114 ± 25 % baseline PS) and 6 ± 2 (57 ± 24% baseline PS), respectively. Only a single advice on PS was provided following PEEP changes.

## Conclusions

These initial results indicate that Beacon Caresystem responds appropriately to over- and under-support.

## Grant Acknowledgment

DSK and SER are minor shareholders and perform consultancy for Mermaid Care.

